# Supplementary material for: Space-time monitoring of groundwater fluctuations with passive seismic interferometry
Source: Nat Commun. 2022 Aug 8;13:4643. doi: 10.1038/s41467-022-32194-3 (PMC9360030; doi:10.1038/s41467-022-32194-3)
Supplement: Supplementary file 1 — Supplementary Information [file 41467_2022_32194_MOESM1_ESM.pdf]

Supplementary Information for

Space-Time Monitoring of Groundwater Fluctuations with Passive Seismic Interferometry

S. Mao *et al.*

This PDF file includes:

Supplementary Notes 1–4
Supplementary Figures 1–6
Supplementary References

Note 1. Structures of the groundwater system in the Costal Los Angeles Basins (CLAB)

The study area (see, main text Figure 1) is located in Los Angeles (LA) metropolitan area in southern California, on the west coast of the contiguous United States. This area consists of a number of groundwater basins (main text Figure 1a, primarily including the Santa Ana Basin, LA Central Basin, LA West Coast Basin, and San Gabriel Basin), which can be collectively referred to as the Coastal LA Basins (CLAB)[1]. The shallow subsurface of these basins is filled with unconsolidated Quaternary-age sediments, mainly from marine or alluvial sources and partly from the erosion of nearby mountains. The aquifer systems extend to a few hundred meters below the surface (up to about 700 m in Santa Ana Basin[2]). With great water storage capacity, these groundwater reservoirs provide 40% of local water supply in normal years and over 60% in dry years.

CLAB are mainly bounded by mountains and faults (main text Figure 1a), including the San Gabriel Mountains to the north, the Hollywood Fault on the northeast, the Palos Verdes Hills to the southwest, and the Whittier Fault to the Southeast. One notable boundary is the Newport-Inglewood Fault (NIF) that separates the LA Central Basin and LA West Coast Basin. This strike-slip fault consists of a series of discontinuous faults and folds that obstruct groundwater flow at depth between the two basins. The groundwater basins are also partitioned by administrative boundaries: the Santa Ana Basin is adjudicated to the Orange County Water District, the LA Central and West Coast Basins to the Water Replenishment District of Southern California, and the San Gabriel Basin to San Gabriel Valley Municipal Water District. Notably, the boundary between the LA Central and Santa Ana Basins is a pure municipal border between the LA County and the Orange County, through which the groundwater flow is possible.

The study area has a Mediterranean climate with dry summers and some storms in winter. In dry summers, heavy pumping from groundwater wells is common in this area for local water supply. As a result, the groundwater storage in the study area shows strong seasonal variations. From previous geodetic studies, the seasonal groundwater fluctuations can cause up to 60 mm annual uplift and subsidence of the surface[3-7]. The primary tectonics in this area include: (1) about 4 mm/yr contraction across this region related to the transpressional Big Bend of the San Andreas Fault[4] and (2) motions associated with strike-slip faults, e.g., ~1 mm/yr along NIF[8]. These tectonic motions are relatively small compared to the seasonal deformation driven by groundwater fluctuations.

The groundwater system in CLAB is characterized by multi-scale, highly-nonuniform structures. Figure 3-2 of supplementary reference 2 shows schematically the cross-section of the groundwater basins, comprising lenses of aquifers (blue area in Figure 3-2 of supplementary reference 2, which is the major water-bearing formations composed of sands and gravels with high permeability and low compressibility) sandwiched among lenses of aquitards (beige area in Figure 3-2 of supplementary reference 2, composed of silt and clays bearing little water with low permeability and high compressibility). The southwest portion of the Santa Ana and LA Central Basins (in Figure 1b of the main text, or the left portion in Figure 3-2 of supplementary reference 2) is featured as a 'confined zone', which contains aquifers confined by thick, laterally-continuous aquitards (clays and silts). These relatively impermeable layers retard the vertical flow of groundwater. In contrast, the northeast portion (in Figure 1b of the main text, or the right portion in Figure 3-2 of supplementary reference 2), is featured as an 'unconfined zone' (or forebay), where groundwater can migrate vertically in

the coarser-grained aquifers relatively freely and the water surface is connected to atmospheric pressure. Aquifers in confined to semi-confined conditions also exist in the San Gabriel Valley. Corresponding to the seasonal fluctuations of groundwater level and, thus, changes in pore pressure, significant deformation has been observed on Earth surface above the confined aquifers in Santa Ana, LA Central and San Gabriel Basins[3-7] (because of the highly compressible clay contents), but not in the unconfined zone (because of the low compressibility of sands and gravels).

Note 2. Amplitudes of $\Delta v/v$ seasonal variability

To retrieve the amplitude of seasonal $\Delta v/v$ fluctuations, we take the $\Delta v/v$ time series on each spatial grid in the study area. For each time series, firstly the long-term change is subtracted from the original $\Delta v/v$ (Supplementary Fig. 1a), then a sinusoidal fitting (with periods of one year and half year) on the seasonal residual using least square optimization is applied (Supplementary Fig. 1b). We use the amplitude of the sinusoidal fitting on each grid as the magnitude of seasonal variability at different locations. Note that the seasonal amplitude does vary from year to year, but only the temporally averaged amplitudes are used (for the seasonal $\Delta v/v$ map in Figure 3 in the main text, seasonal amplitudes over 2000-2011 are used to best compare with InSAR seasonal amplitudes[3] averaged over 1992-2011).

Note 3. The relationship between annual rainfall- and pumping-induced $\Delta v/v$

We calculate the basin-scale temporal trends by averaging the $\Delta v/v$ time series over spatial grids within each of the three basins (Santa Ana, LA Central and San Gabriel Basins) (Supplementary Fig. 3a-c, respectively). It shows that the long-term trends of groundwater storage in these three basins have been quite different over the past two decades: In Santa Ana Basin the temporal fluctuations exhibit both increasing and decreasing episodes, and they periodically recover and balance in the long term. In the San Gabriel and LA Central Basins, however, the long-term fluctuations are dominated by decreasing trends. The spatial distributions of the cumulative changes are depicted using 0.2-0.8 Hz (main text Figure 4, at ~700 m depth), and 0.2-2.0 Hz (Supplementary Fig. 4, at ~350 m depth).

To better understand why opposite signs of groundwater change have occurred in the adjacent basins (in particular, slight increase of groundwater in Santa Ana and decline in LA Central basins), we consider the two primary seasonal factors, precipitation and well pumping, that contribute to groundwater changes in every year. These two components are highly seasonal in the study area: rainfall mainly occur in winters and well pumping in summers. From the $\Delta v/v$ time series for each basin, we extract the rainfall-induced decrease of $\Delta v/v$ (i.e., increase of groundwater) from the difference between valleys and peaks from October 1st to the following May 31st in each water year, and the pumping-induced increase of $\Delta v/v$ (or decrease of groundwater) from the difference during May 1st to October 31st. We show the relationships between these two seasonal components during 2000-2020 in Figure 4b-d of the main text, where the vertical axes denote the pumping-induced decreases of each year and the horizontal axes rainfall-induced increases. To downweigh the effect of a few extreme climate events, the decrease of $\Delta v/v$ are obtained by averaging over every three years (backwards). The three-year averaging scheme is chosen following the practice by which Orange County Water District determines the pumping quota[9] (three is a somewhat arbitrary choice to assess recharge over a slightly longer period). Figure 4b of the main text shows that in the Santa Ana Basin the rainfall- and pumping-induced changes are strongly correlated in a linear relationship (p-value = 0.0017), and the slope of the best fitting line is close to 1. In the LA Central and San Gabriel

Basins (see, main text Figure 4c and d, respectively), however, there is little evidence of linear relationship (p-values both above 0.65), and more data points located on the upper-left triangle that correspond to years of overdraft. The implications of this analysis are discussed in the main text.

In some years during the study period, the artificial injection of water through recharge basins or at injection wells can be a minor source of groundwater recharge (apart from rainfall). We note that isolating the effect of artificial injection from the rainfall- and pumping-induced changes does not alter the (positive or negative) distance of the scattered points to the y=x line in the scatter diagrams (in Figure 4b-d of the main text), and thus does not affect the interpretations regarding the relative amplitudes of rainfall- and pumping-induced changes. So in the aforementioned analysis we did not isolate this component.

Note that in Figure 4 of the main text and Supplementary Fig. 4, the spatial patterns of cumulative $\Delta v/v$ changes are mainly associated with changes in aquifer storage. We did not observe spatial features related to oil and gas operations in this region[3-6], which are often in bullseye shape (less than a-few-kilometer scale), likely due to the limited spatial resolution of $\Delta v/v$ measurements.

Note 4. LA West Coast Basin

It is also worth noting the cyan-blue area in Figure 4 of the main text, which coincides with the shape of the LA West Coast Basin. Being close to the ocean, the velocity decrease in this area might be caused by the increasing (mass) density of the underground fluid content due to seawater intrusion; it is also possible to be caused by the increase of groundwater volume due to the artificial injections in this basin over the past years[10]. Using $\Delta v/v$ alone cannot distinguish between these scenarios. But the coincident shape and locations of $\Delta v/v$ decrease and the LA West Coast Basin calls for further investigations, for instance measurements of salt and nutrient concentrations, or imaging electrical resistivity[11], to quantify the effect of groundwater storage increase or seawater intrusion.

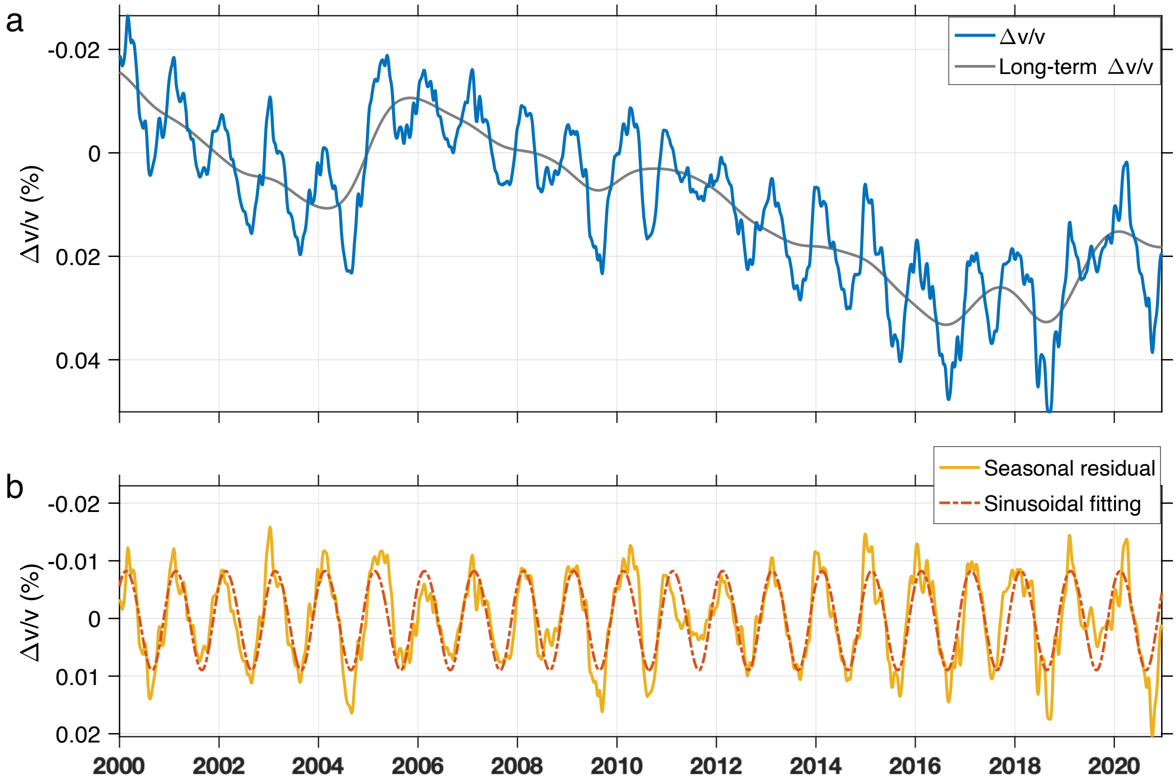

Supplementary Fig. 1 **Retrieval of the amplitude of seasonal variabilities. a** The time series of original (in blue) and long-term (in grey) *Δv/v*. **b** The seasonal residual of *Δv/v* (bold yellow line) and the sinusoidal fitting (dashed orange line) based on least-square optimization. The fitting function contains sinusoids of one-year and half-year periods.

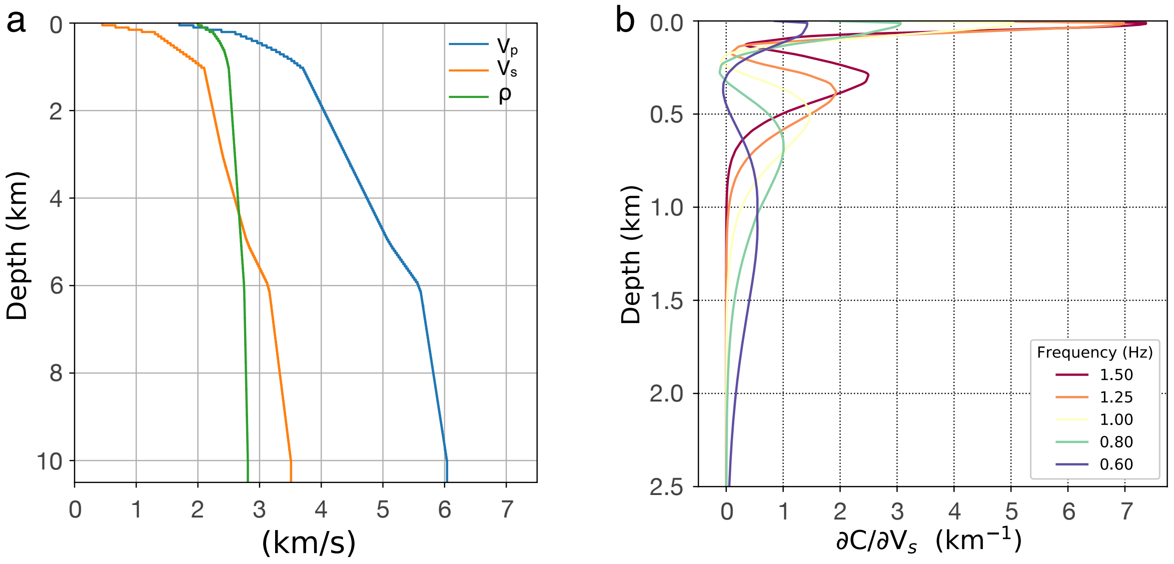

Supplementary Fig. 2 **Depth sensitivity kernels of seismic waves in different frequencies. a** The averaged 1-D velocity model in Los Angeles basin[12]. **b** Depth sensitivity kernels of Rayleigh waves based on the velocity model in **a**[13].

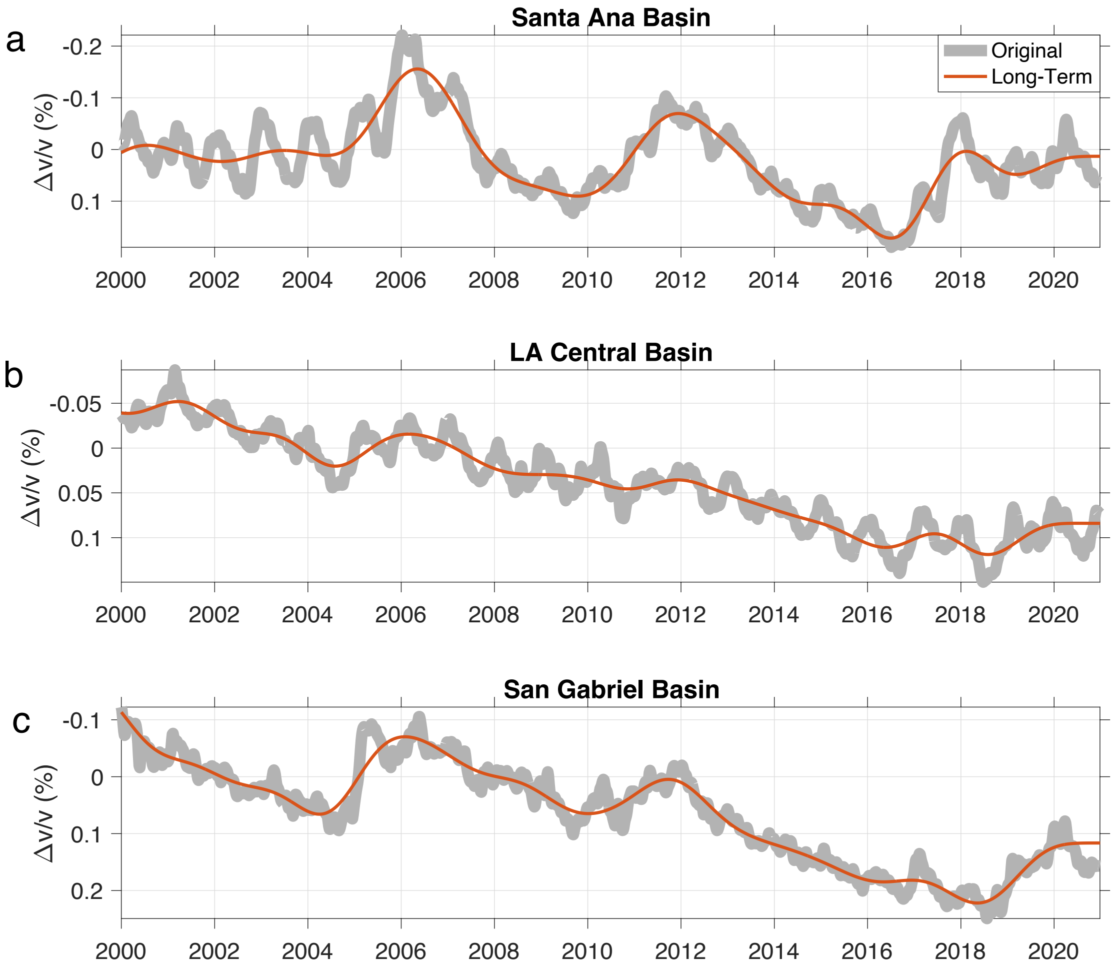

Supplementary Fig. 3 **The basin-wide long-term variations of *Δv/v*.** The time series of *Δv*/v are averaged over all the spatial grids within **a** Santa Ana Basin, **b** LA Central Basin, and **c** San Gabriel Basin. The grey shades denote the original *Δv/v*, and the orange lines denotes the low-pass filtered long-term *Δv/v*.

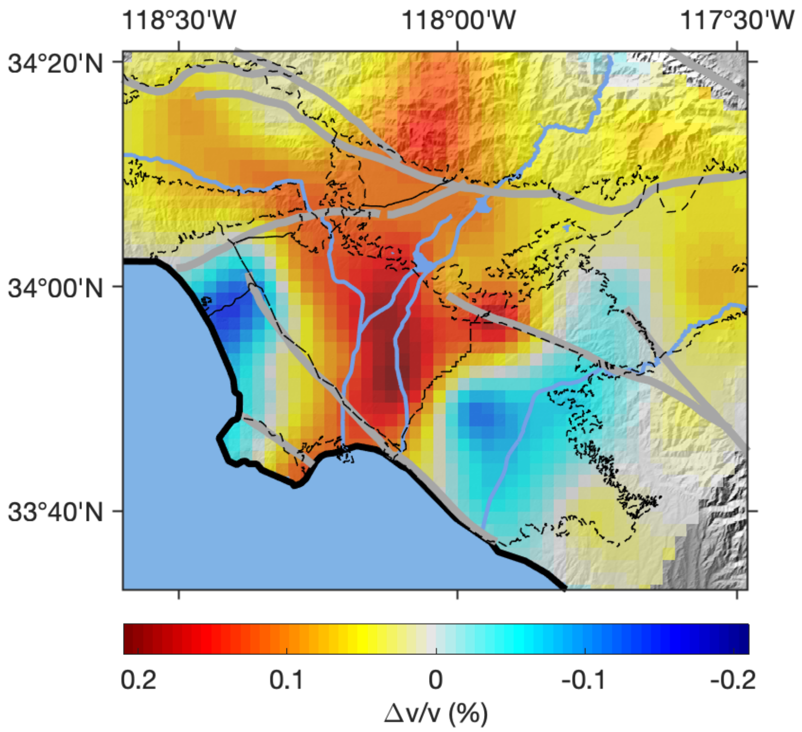

Supplementary Fig. 4 **The map of accumulated *Δv/v* from 2000-2020 measured in 0.5-2.0 Hz.** Colder colors associate with cumulative increases of groundwater and warmer colors cumulative declines. The spatial patterns in this map (corresponding to ~350 m depth) are in general similar to those in Figure 4a of the main text (corresponding to deeper depth at ~700 m), with slight recovery in groundwater storage shown in Santa Ana but depletion in LA Central and San Gabriel Basins.

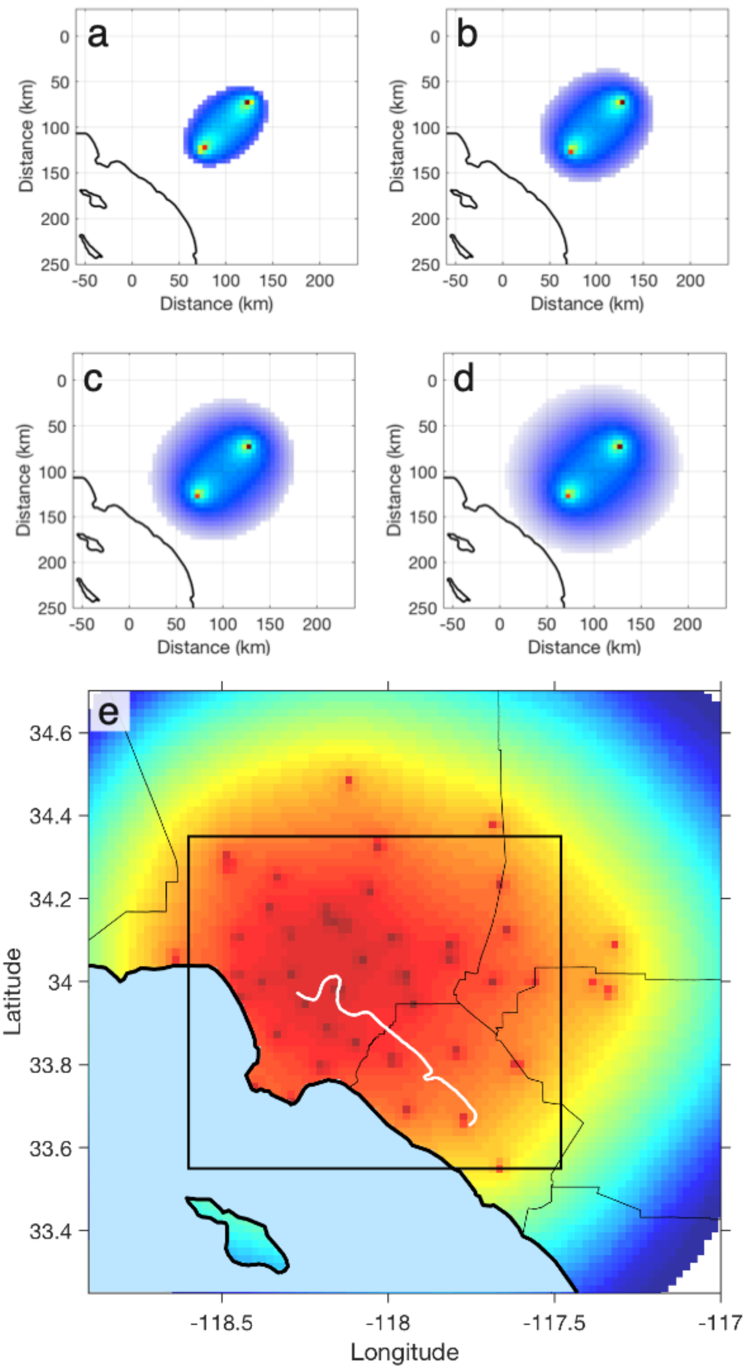

Supplementary Fig. 5 **Illustration of coda-wave sensitivity kernels. a-d** Examples of coda-wave sensitivity kernels at increasing travel times for a single station pair, derived under radiative transfer theory[14]. They show that coda waves with different travel-times pick up the velocity changes in different areas. **e** Sum of all kernels from all station pairs used in this study. The black box denotes the area with adequate sensitivities, in which the inverted *Δv/v* is analyzed in this study.

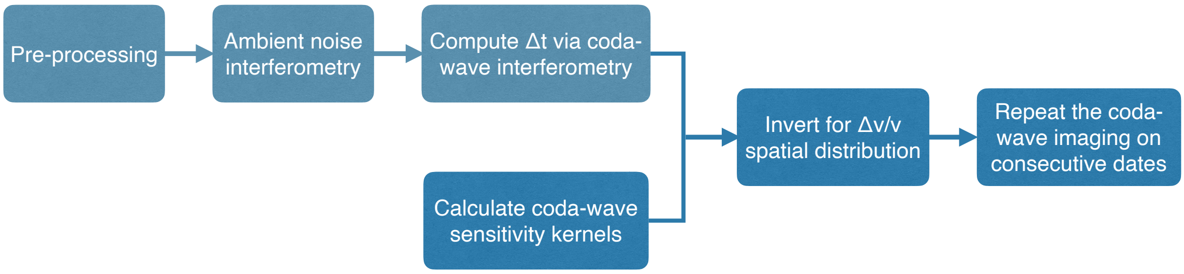

Supplementary Fig. 6 **Workflow of the data-processing for space-time seismic interferometry.**

Supplementary References

1. Goldrath, D., Fram, M. S., Land, M. & Belitz, K. Status of Groundwater Quality in the Coastal Los Angeles Basin, 2006: California GAMA Priority Basin Project. *US Department of the Interior, US Geological Survey* (2012).

2. Orange County Water District (OCWD), Groundwater Management Plan 2015 Update (2015). https://www.ocwd.com/what-we-do/groundwater-management/groundwater-management-plan

3. Riel, B., Simons, M., Ponti, D., Agram, P., & Jolivet, R. Quantifying ground deformation in the Los Angeles and Santa Ana Coastal Basins due to groundwater withdrawal. *Water Resources Research*, 54(5), 3557-3582 (2018).

4. Bawden, G. W., Thatcher, W., Stein, R. S., Hudnut, K. W., & Peltzer, G. Tectonic contraction across Los Angeles after removal of groundwater pumping effects. *Nature*, 412(6849), 812-815 (2001).

5. Lanari, R., Lundgren, P., Manzo, M., & Casu, F. Satellite radar interferometry time series analysis of surface deformation for Los Angeles, California. *Geophysical Research Letters*, 31(23) (2004).

6. Argus, D. F., Heflin, M. B., Peltzer, G., Crampé, F., & Webb, F. H. Interseismic strain accumulation and anthropogenic motion in metropolitan Los Angeles. *Journal of Geophysical Research: Solid Earth*, *110*(B4) (2005).

7. Houlie, N., Funning, G. J., & Bürgmann, R. Use of a GPS-derived troposphere model to improve InSAR deformation estimates in the San Gabriel Valley, California. *IEEE Transactions on Geoscience and Remote Sensing*, *54*(9), 5365-5374 (2016).

8. Wright, T. L. Structural Geology and Tectonic Evolution of the Los Angeles Basin, California: Chapter 3: PART 1 (1991).s

9. Water Replenishment District (WRD) of Southern California Regional Groundwater Monitoring Report 2019-2020, Central and West Coast Basins, Los Angeles County, California (2021). https://www.wrd.org/reports/regional-groundwater-monitoring-report

10. Goebel, M., Pidlisecky, A., & Knight, R. Resistivity imaging reveals complex pattern of saltwater intrusion along Monterey coast. *Journal of Hydrology*, *551*, 746-755 (2017).

11. Small, P., Gill, D., Maechling, P. J., Taborda, R., Callaghan, S., Jordan, T. H., Olsen, K. B., Ely, G. P. & Goulet, C. The SCEC unified community velocity model software framework. *Seismological Research Letters*, *88*(6), 1539-1552 (2017).

12. Herrmann, R. B. Computer programs in seismology: An evolving tool for instruction and research. *Seismological Research Letters*, 84(6), 1081-1088 (2013).

13. Margerin, L., Planès, T., Mayor, J., & Calvet, M. Sensitivity kernels for coda-wave interferometry and scattering tomography: theory and numerical evaluation in two-dimensional anisotropically scattering media. *Geophysical Journal International*, 204(1), 650-666 (2016).
